# Successful Treatment of Transient Central Diabetes Insipidus following Traumatic Brain Injury in a Dog

**DOI:** 10.1155/2019/3563675

**Published:** 2019-04-22

**Authors:** Catriona Croton, Sarah Purcell, Andrea Schoep, Mark Haworth

**Affiliations:** Small Animal Hospital, University of Queensland, Gatton 4343, Australia

## Abstract

An 11-year-old female spayed Maltese presented comatose, half an hour after vehicular trauma, and was treated for traumatic brain injury and pulmonary contusions. The dog developed severe hypernatremia within six hours of presentation, which responded poorly to the administration of five percent dextrose in water. As central diabetes insipidus was suspected, desmopressin was trialled and resolution of hypernatremia was achieved six days later. Transient trauma-induced central diabetes insipidus has been described previously in two dogs; in the first, serum sodium concentrations were evaluated three days after injury and the other developed hypernatremia seven days after injury. To the authors' knowledge, this is the first report of rapid onset, transient, and trauma-induced central diabetes insipidus in a dog that encompasses the complete clinical progression of the syndrome from shortly after injury through to resolution.

## 1. Introduction

Central diabetes insipidus (CDI) is due to the partial or complete lack of secretion of antidiuretic hormone (ADH) from the posterior pituitary, resulting in a hypotonic polyuria [1]. CDI may occur secondary to traumatic brain injury (TBI) and when coupled with injuries preventing compensatory polydipsia, severe dehydration and hypernatremia can occur [2]. In people with moderate to severe TBI, the incidence of trauma-induced CDI has been estimated at 26-28% [3, 4] and carries a poor prognosis [4, 5]. Moreover, there is an increase in mortality rate if the CDI develops within the first three days following injury or if the hypernatremia is severe [4, 5]. Although there are no comparable studies published in the veterinary literature, in a recent paper, all TBI dogs with a serum sodium (sNa) >160 mmol/L were nonsurvivors [6].

Transient trauma-induced CDI has been described previously in a female spayed dog referred three days after injury, by which time the hypernatremia was already present [7]. In addition, a case of suspected traumatic panhypopituitarism in an intact female dog has also been described; however, the hypernatremia occurred seven days after injury [8]. This case report describes the successful treatment of a dog with rapid onset, trauma-induced CDI, from shortly after trauma through to the resolution of laboratory abnormalities and clinical signs 8 days later.

## 2. Case Presentation

### 2.1. Day 1

An 11-year-old, 6.6 kg, female spayed Maltese, presented comatose to the University of Queensland Veterinary Teaching Hospital within 30 minutes of blunt force trauma after being hit by a car. The dog was previously well with no current medications. Initial physical examination revealed the dog to be laterally recumbent and comatose, with bilateral pin-point pupils and an absent menace response. The oral mucous membranes were cyanotic and the dog rapidly progressed to respiratory arrest. The heart rate was initially 60 beats per minute (bpm). Unfortunately, a blood pressure reading was not recorded at this time. There was haemorrhage from the mouth with trauma evident to the oral mucosa.

A venous blood gas performed at presentation showed a mixed acidosis (pH 6.97 [reference interval: 7.35-7.44], lactate 12.4 mmol/L [reference: <2 mmol/L], pCO_2_ 44 mmHg [reference interval: 33.6-41.2 mmHg]), and hyperglycaemia of 24.8 mmol/L [reference interval: 3.3-6.8 mmnol/L]. The sNa was in the normal range at 138 mmol/L [reference interval: 135-153 mmol/L].

An intravenous (IV) catheter was placed and 3 mg alphaxalone was given IV to permit intubation and manual intermittent positive pressure ventilation with 100% oxygen. A 20 ml/kg IV bolus of lactated Ringer's solution (LRS) was administered, followed by a 4 ml/kg bolus of 7% hypertonic saline (HTS) and an infusion of 0.5g/kg of mannitol over 20 minutes. LRS was subsequently continued at approximately 10 ml/kg/hr for one hour and then reduced to 5 ml/kg/hr. Analgesia was provided with fentanyl at 2 ug/kg IV bolus for three sequential boluses, followed by a constant rate infusion (CRI) at 4 ug/kg/hr.

Once spontaneous ventilation was noted, extubation was achieved and treatment continued in an oxygen cage. Neurological status at this time was improving, however, the dog was still obtunded with ongoing bilaterally miotic pupils and an absent menace response. The heart rate had increased to 116 bpm with strong femoral pulses and a systolic blood pressure of 180 mm Hg as measured by doppler. The respiratory rate was 60 breaths per minute with bilaterally harsh lung sounds in all fields. Chest radiographs were performed which demonstrated changes consistent with pulmonary contusions in the right cranial and left caudal lung fields. The dog was assessed as having a TBI with pulmonary contusions.

**Figure 1:**
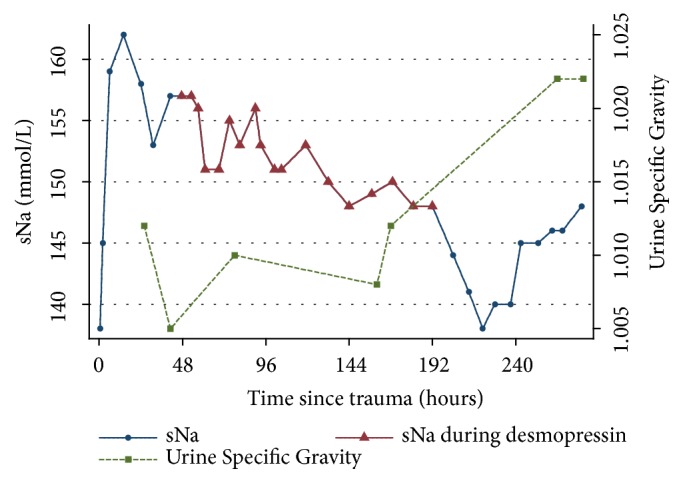
Serum Na concentrations are shown on the left vertical axis, with red triangles showing when desmopressin treatment started and ended, and urine specific gravity on the right vertical axis (generated using Stata v14 (StataCorp LP, College Station, Texas)).

Approximately two hours after presentation and following initial treatment, a repeat venous blood gas showed the lactate had decreased to near normal at 2.8 mmol/L, with a pH of 7.34, and the hyperglycaemia had resolved (glucose 6.4 mmol/L). The dog's neurological status improved slightly, though some agitation was subsequently noted. A 0.01 mg/kg IV bolus of acepromazine was administered to address this. Five and a half hours after presentation, a venous gas showed a markedly increased sNa of 159 mmol/L (Figure 1). Other blood work remained unremarkable.

The following morning, fourteen hours following presentation the sNa was measured to be 162 mmol/L. A free water deficit of 480 ml was calculated, and her IV fluids were changed to five percent dextrose in water (D5W) at 40 ml/hr, concurrently with LRS at 25 ml/hr. The goal was to replace the free water deficit over 12 to 24 hours with a decrease of sNa of 0.5 mmol/L/hour. Despite this, 24 hours after presentation and 12 hours following free water commencement, the sNa had only decreased by 4 mmol/L to 158 mmol/L. The urine specific gravity (USG) was 1.012 concomitantly (Figure 1).

The dog was noted to have improved consciousness, but was intermittently vocalising. A head tilt to the right and rolling to the left when handled was also noted. The menace response remained bilaterally absent and there was absent conscious proprioception in both forelimbs and delayed in the hindlimbs. Withdrawal was present in all limbs. The dog was noted to be urinating large volumes frequently and refused to eat or drink. Harsh lung sounds were evident bilaterally, with an SpO2 of 94% on room air and 98% whilst receiving cage oxygen. Due to ongoing vocalisation, phenobarbitone at 2 mg/kg slow IV was commenced every 12 hours for sedation.

### 2.2. Day 2

Approximately forty hours after presentation, the dog's mentation had markedly improved as evidenced by reduced anxiety and vocalisation. The dog was responsive to sound and was able to ambulate with assistance. Oxygen supplementation was discontinued as pulmonary function had normalised, and the fentanyl CRI was reduced to 2 ug/kg/hr. Nutritional support was initiated at this time in the form of Hills a/d slurry via syringe with a total volume of 10 ml given on this day.

Despite ongoing parenteral free water supplementation, the sNa remained elevated at 157 mmol/L, with a USG of 1.005. This poor response to free water supplementation prompted desmopressin (DDAVP) administration, one drop of 4 *μ*g/ml solution into the conjunctival sac. Free water administration continued unaltered. Measurement of sNa 12 hours later revealed no change, remaining steady at 157 mmol/L. Therefore, a second dose of one drop DDAVP was administered conjunctivally. Almost no effect was noted as six hours later; the sNa was 156 mmol/L. At this time the desmopressin dose was increased to two drops every 12 hours. Three hours later, sNa was 151 mmol/L with concurrent D5W at 40 ml/hr and LRS at 25 ml/hr.

### 2.3. Day 3

The dog's neurological status continued to improve. By the third day the menace response had returned in the left eye; there was reduced rolling and circling, and return of conscious proprioception in both forelimbs was noted. Despite the improvement in neurological status the dog developed intermittent head pressing, displayed ongoing subjective polyuria, and was also noted to be lame in the left foreleg, which continued throughout her hospital stay. A transient increase in rectal temperature was also noted (40.2°C); however, this normalised following active cooling with a fan and was thought most likely secondary to anxiety. Maropitant was administered at 1 mg/kg subcutaneously every 24 hours for suspected nausea (ptyalism) and ongoing inappetence. The dog subsequently began to eat 20 mls of Hills a/d slurry every four to six hours. Water was offered every four hours. LRS and fentanyl were discontinued, and D5W was increased to 50 ml/hr. She was continued on two drops of DDAVP in the conjunctival sac every 12 hours.

### 2.4. Day 4

Despite this increase in D5W and DDAVP, during the fourth day the dog's sNa had increased again to 156 mmol/L and DDAVP was subsequently increased to three drops every 12 hours. Urine specific gravity was recorded to be 1.010 at the commencement of day 4. A mild hypokalaemia of 3.3 mmol/L (reference range: 3.4–4.9 mmol/L) was noted and LRS with 40 mmol/L KCl was recommenced at 10 ml/hr and the D5W was reduced to 40mls/hr. Four hours after the increase in DDAVP the sNa dropped to 151 mmol/L.

### 2.5. Day 5

By the fifth day the sNa was between 151 and 153 mmol/L and frequent urination continued. The DDAVP was decreased to two drops every 12 hours, and the fluids remained unchanged.

### 2.6. Day 6 to 8

On the sixth day sNa was 148 mmol/L. The dog became more alert and aggressive, which was consistent with her temperament prior to the TBI. She was starting to lick slurry from a bowl in addition to the syringe feeding commenced earlier. Over the following two days, her sNa remained between 148 and 150 mmol/L with a K of 4.4-4.8 mmol/L and a USG of 1.008. The clinical status remained unchanged and treatment was continued with 2 drops of DDAVP every 12 hours, and the D5W was reduced to 10 ml/hr. The USG increased slightly to 1.012 by day 8.

### 2.7. Day 9 to 12

In the morning of day 9, the dog was ambulatory, although bumping into objects. The sNa was 144 mmol/L, and the DDAVP was therefore discontinued. The following day, the sNa was 140 mmol/L with a K of 6.2 mmol/L. The blood pH was 7.21, bicarbonate 11.2 mmol/L, pCO_2_ 28 mm Hg, and base excess -15.2 mmol/L. The urea was 12.40 (reference interval: 3.40–10.80 mmol/L). She was given sodium bicarbonate (8.4%), 10 mmol, diluted in 50 ml of saline IV over three hours. A urine sample collected by cystocentesis showed cocci on sediment exam and amoxicillin clavulanic acid (12.5 mg/kg) was initiated subcutaneously once daily. The sNa for that day was 146 mmol/L and the K dropped to 5.1 mmol/L.

### 2.8. Day 13

The dog had improved mentation with no head pressing or circling. Although she was ambulatory without ataxia, she would follow walls when walking. Her vision appeared reduced. The USG was 1.022 with a sNa of 148 mmol/L. She was discharged home on day 13 with oral amoxicillin clavulanic acid.

### 2.9. Follow Up

The dog returned two days later for a recheck, at which time her owner reported almost normal mentation. A repeat sNa was 152 mmol/L with a K of 4.8 mmol/L. She had an ongoing lameness of 4/5 in her left foreleg with no distinct foci of pain on palpation of the limb. Her aggressive behaviour limited the musculoskeletal examination.

During a final recheck 10 days later, the owner reported that her personality and behaviour had returned to what they had been before the accident. Given her ongoing left forelimb lameness, she was anaesthetised the following day after a preanaesthetic blood screen, in which her sNa was 143 mmol/L. Radiographs were taken of both forelimbs and showed bilateral severe osteoarthritis of her elbows, with the left being more severe than the right. The persistent lameness was likely from osteoarthritis exacerbated by trauma.

## 3. Discussion

Diabetes insipidus is categorised as either the central form, in which there is a lack of secretion of ADH (antidiuretic hormone, vasopressin), or the nephrogenic form, with a lack of renal response to ADH [1]. ADH is secreted from the posterior pituitary gland in response to either increased plasma osmolality or decreased arterial pressure and acts on V1, V2, and V3 receptors [9]. The main physiological effect of ADH is through the V2 receptors which act on the specific water channel proteins, aquaporins, in the luminal wall of the collecting duct in the kidney [9]. In CDI, the lack of ADH, either partial or complete, results in downregulation of the aquaporins causing free water loss in the urine. In an otherwise clinically well patient, this hypotonic polyuria would be compensated for by polydipsia. However, this is often not possible in a patient with TBI due to either impaired consciousness or an inability to drink [2] resulting in hypovolaemic hypernatremia. To complicate the clinical picture, hypernatremia itself can cause neurological signs including lethargy, ataxia, weakness, seizures, and coma [10, 11], and the signs may depend more on the rapidity of the rise in sNa, rather than the magnitude [12]. These changes may contribute to the high mortality rate seen in people with acute traumatic CDI and serial monitoring to ensure early detection of such derangements is recommended [2].

Mortality rates for dogs with head trauma have been estimated at 18-24% [13], and in dogs with severe blunt trauma, those with TBI have significantly decreased survival [14]. The incidence of CDI in people with TBI is estimated at 15-28%, but this can vary widely depending on the inclusion and diagnostic criteria used, and it has a mortality rate of 69% [15]. People with early onset of CDI developing within three days of trauma have a poorer prognosis with 86% mortality [5]. The dog in this case report displayed changes consistent with trauma-induced CDI within 7 hours of trauma. This is the first case report of early onset trauma-induced CDI in a dog that the author is aware of.

It is generally accepted that the diagnostic criteria for acute traumatic CDI in people is a hypotonic polyuria with plasma hypernatremia and hyperosmolality, but there is a lack of consensus on the exact cut-off point for these. One diagnostic criteria commonly used is polyuria defined as urine volume > 2 ml/kg/hr, urine osmolality below 300 mosm/kg, and increased plasma osmolality > 300 mOsm/kg [15, 16]. In the acute phase in people, sNa > 143 mmol/l with polyuria is considered suggestive of CDI [16]. In dogs, although guidelines for the acute traumatic CDI have not been agreed upon given the few cases reported, classification of CDI after trans-sphenoidal surgery is defined as a USG ≤ 1.005 or sNa ≥ 160 mmol/L [17].

The dog in this case report had a peak sNa of 162 mmol/L at 12 hours following trauma, from an initial concentration of 138 mmol/L (see Figure 1). Causes of increasing sNa can be attributed to a relative loss of free water or sodium gain [18]. Hyperglycaemia causes an osmotic diuresis with free water loss; however, it is unlikely that this was a contributing factor in this dog, as the hyperglycaemia resolved rapidly with initial resuscitation. Hypertonic fluid administration can cause an increase in impermeant solute or a subsequent loss of free water. During resuscitation this dog received a single bolus of 25 ml of 7% hypertonic saline and a single bolus of mannitol of 0.5 g/kg slow IV infusion. Both may contribute to increases in sNa by solute gain or water loss, respectively. However, this effect would be expected to manifest more rapidly, as these medications were given intravenously at standard doses, and the effects of these hyperosmotic agents on sNa would also be expected to resolve relatively rapidly following administration. The dog also received IV LRS at greater than maintenance rates. This slightly hypotonic crystalloid would have also provided additional free water with a sodium content of 131 mmol/L (Baxter). During the first 12 hours the dog received approximately 455 ml of LRS which represents a free water volume of approximately 86 ml relative to the peak sNa. Further, despite administration of IV D5W at a rate considered sufficient to correct the sodium over 12 to 24 hours, the sNa remained high at 157 mmol/L with a USG of 1.005. Additionally, the sNa remained elevated for several days despite ongoing free water administration to address this imbalance.

Polyuria associated with the hypernatremia was observed in this dog. It is an acknowledged limitation of this case report, however, that no urinary catheter was placed to quantify urine output (UOP). Other causes of polyuria in the TBI patient include excessive fluid administration, hyperosmolar fluid administration (such as mannitol or HTS), hyperglycaemia, and the administration of diuretics [19]. Although both mannitol and HTS were given, only a single dose of both was administered during initial stabilisation. The initial hyperglycaemia normalised during this time and was unlikely to contribute. The total LRS dose administered during the first 12 hours was in excess of the maintenance rate and the addition of D5W the following day may have also contributed to the subjective polyuria. Fluid therapy was not withheld at any stage and this may have obscured polyuria associated with CDI.

Computed tomography (CT) and magnetic resonance imaging (MRI) were not performed for diagnosis in this case due to the patient responding clinically to treatment and cost. Fractures in the region of the hypothalamus and pituitary gland have previously been reported in dogs with traumatic CDI [7, 8]. However, absence of these changes does not rule out traumatic CDI and it has been previously reported that 6% of people with trauma-induced CDI had no changes in their cerebral CT/MRI [20]. Imaging is usually only indicated in dogs with TBI when there is a poor response to treatment or clinical deterioration after initial improvement [21]. Another possible limitation is the lack of further hormonal tests, including serum cortisol, thyroid stimulating hormone, and free serum thyroxine, which may have impacted the sNa concentrationss. However, any coexisting injury to the anterior pituitary or hypothalamus resulting in reduced serum thyroid hormone or central hypocortisolism would be expected to cause a decrease in sNa rather than a rise [1, 22]. The measurement of serum ADH does not appear to be useful in people [2].

Traumatic CDI was suspected in this case, given the rapid onset of hypernatremia soon after trauma combined with polyuria and hyposthenuria and due to the persistent hypernatremia despite the administration of free water. Therefore, a desmopressin (DDAVP) challenge was performed. DDAVP (1-deamino-8-D-arginine vasopressin) is a synthetic analogue of vasopressin and has a strong affinity for renal V2 receptors which exert an antidiuretic effect with minimal pressor activity [1, 23]. Response to treatment is assessed in people by an increased urine osmolality and decreased serum osmolality [2]. In a previously reported case of transient traumatically induced CDI in a dog, this occurred within 4 hours of vasopressin administration [7]. In the veterinary emergency setting, a USG rather than osmolality will likely be performed. Serum and urine osmolality were not assessed in this dog; however, a decrease in sNa (157 mmol/L to 151 mmol/l) and an increase in USG (1.005 to 1.010) were seen after DDAVP administration. The duration of action of DDAVP varies, and it is therefore dosed to effect. The dose and dosage interval of DDAVP should be kept to the minimum needed to resolve polyuria and slowly reduce sNa by no more than 0.5 mmol/L/h or 10-12 mmol/L/24 h [2]. The dog in this case report was given DDAVP every 12 hours, with the dose increased from 1 drop initially to 3 drops until the response was considered appropriate. There was an overall gradual decrease in sNa over the first 7 days and a corresponding increase in USG initially. This response following DDAVP administration makes CDI most likely. A more marked increase in USG was only seen once fluid therapy was weaned around day 8 to 9. Retention of free water relies not only on the presence of aquaporins, but also on the medullary concentration gradient which may have been compromised by medullary wash-out due to the high fluid rates the dog was receiving. Free water volume and rate were deescalated in concert with DDAVP after the dog's sNa was observed to be improving and oral water intake had increased. Another method used to diagnose CDI is the water deprivation test or modified water deprivation test [24]. This test is not appropriate in the acute stage of trauma-induced CDI and was therefore not performed.

There have been previous reported cases of CDI following TBI in dogs. The first reported case was in a female spayed dog who presented three days after injury with severe hypernatremia which responded to vasopressin administration [7]. Vasopressin was discontinued 28 days after injury as the hypernatremia had resolved. In another case report, an intact female dog with hypothalamic-pituitary axis deficiency following TBI responded to DDAVP, but was euthanased on day 8 of treatment due to a poor prognosis and therefore it is unknown if resolution would have occurred [8]. A further case of CDI was reported in a male intact dog after cardiopulmonary arrest which occurred following a surgical procedure. This dog was treated with DDAVP which was successfully discontinued after two weeks of therapy [25].

The pituitary gland is vulnerable during head trauma due to its location, vascular supply, and delicate structure [26]. Damage from the trauma can be either primary, due to direct mechanical damage at the time of the trauma, or secondary, which occurs following the trauma and includes damage to small vessels and inflammatory oedema [27]. In this case, as the DDAVP was able to be discontinued and the CDI was transient, the cause was more likely secondary. In people with TBI, CDI is often transient, with the majority resolving within days to weeks; however, in a small percentage of people the condition is permanent [28]. Therefore, the clinician needs to be mindful that CDI following trauma in dogs may be transient with long-term therapy not required, which may affect the owner's decision to continue with treatment.

This case reinforces that serial monitoring of neurological status, sNa, and UOP in TBI patients is warranted. Elevations of sNa, or even sNa trending towards the upper limits of the reference range, should be a cause for concern and monitored closely. Early signs of trauma-induced CDI include the production of large volumes of hypotonic urine (USG ≤ 1.005), especially in the presence of a rising sNa and a normal or high plasma osmolality. This highlights the importance of monitoring fluid balance closely in TBI patients, especially in those who are unable to drink and therefore lack the usual compensatory pathway of polydipsia in the event of polyuria. Monitoring in these patients would ideally include the placement of an indwelling urinary catheter with a closed collection system, to permit regular measurement of UOP and USG and allow early detection and quantification of polyuria. In addition, frequent weighing of the patient to aid in detection of fluid imbalance is recommended. Serial monitoring of electrolytes is also warranted as these can be deranged secondary to polyuria. In this case, there was a rise in the sodium within hours of injury, but in people there can be a delay of up to four to ten days [2].

## 4. Conclusion

Despite a poor prognosis in people with rapidly developing CDI following trauma, successful treatment of the dog in this case report demonstrates the possibility of a positive outcome in dogs with trauma-induced CDI. This disease may carry a more favourable prognosis in dogs than has been reported in the human literature. Further investigation into the prognosis of dogs with trauma-induced CDI is required; however, reported cases are rare. This is the first documented case of a dog with rapid onset trauma-induced CDI from presentation through to its resolution. The timely identification of polyuria, hypernatremia, and dehydration in dogs following trauma is important for a successful outcome, particularly in TBI patients who are unable to take oral fluids. Given the high incidence of this disease in people with TBI and the large number of TBIs occurring in small animals, this may be an underdiagnosed disease in the veterinary population.

## Data Availability

No data were used to support this case report.

## Abbreviations

ADH: Antidiuretic hormone
CDI: Central diabetes insipidus
CRI: Continuous rate infusion
D5W: Five percent dextrose in water
IV: Intravenous
LRS: Lactated Ringer's solution
sNa: Serum sodium
TBI: Traumatic brain injury
UOP: Urine output
USG: Urine specific gravity.
